# The arsenic proteome in cancer: Unravelling molecular mechanisms of anticancer drugs

**DOI:** 10.1007/s00775-026-02143-2

**Published:** 2026-04-24

**Authors:** Hongyan Li, Hongzhe Sun

**Affiliations:** Department of Chemistry, the University of Hong Kong, SAR Pokfulam Road, Hong Kong, P.R. China

**Keywords:** Arsenic, Cancer, Mechanism, Metalloproteomics, Proteome

## Abstract

Arsenic is a dual-natured element, functioning as both a carcinogen and a therapeutic agent in cancer treatment. Arsenic trioxide is a clinically approved drug for acute promyelocytic leukaemia, administered either alone or in combination with all-trans retinoic acid. Additionally, arsenic trioxide and organic arsenic compounds, such as darinaparsin, show promising efficacy against various cancers. Given the multi-targeted nature of metallodrugs, a comprehensive understanding of their molecular mechanisms is critical for the rational design of more potent and less toxic therapeutics. In this review, we first outline the structures and activities of key arsenic-based anticancer agents, encompassing both inorganic and organic forms. We then provide a comprehensive overview of proteome-wide identification of arsenic-binding proteins in cancer cells, utilizing metalloproteomic approaches such as immobilized metal affinity chromatography, continuous-flow electrophoresis coupled with inductively coupled plasma mass spectrometry, biotinylated arsenical pull-down, and fluorescence-based metalloproteomics. Subsequently, we discuss how the identified arsenic proteome facilitates elucidation of the molecular mechanisms of arsenic trioxide and darinaparsin from a systems perspective. We also evaluate studies on the cytotoxicity of arsenic compounds at the single-cell level, highlighting their role in improving our understanding of the mode of action of a drug. Finally, we prospect future research direction in this field. This review aims to stimulate further investigation into the molecular mechanisms of arsenic-based drugs at a systems level, guiding the development of more effective and safer arsenic-based therapeutics for cancer treatment.

**Graphical abstract:**

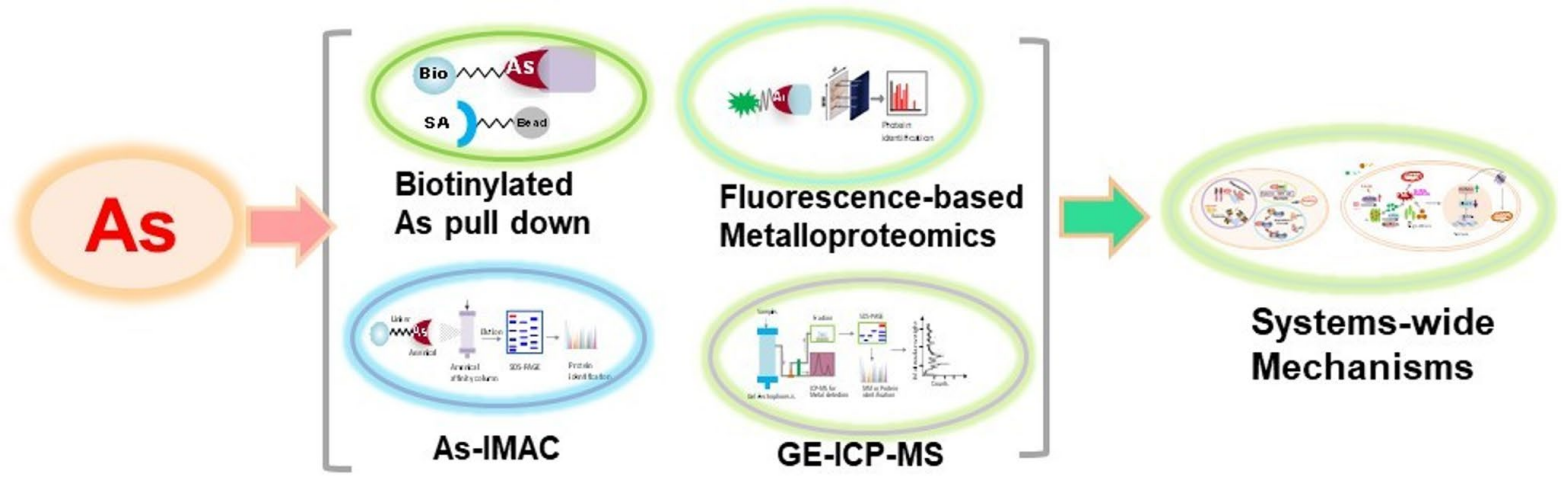

## Introduction

Arsenic has been utilized as a therapeutic agent for over two centuries to treat various ailments. The first metallodrug, Salvarsan, a mixture of 3-amino-4-hydroxyphenyl-arsenic(III) compounds, was developed by Paul Ehrlic in 1910, for the treatment of syphilis and African trypanosomiasis (sleeping sickness). Arsenic trioxide (ATO) and arsenic minerals have been used in traditional Chinese medicine for over 2000 years but fell out of favour due to their toxicity [1, 2]. In the late 19th century, Thomas Flower’s potassium-based arsenic trioxide solution was employed to treat a range of conditions, including leukaemia, Hodgkin’s disease and pernicious anaemia [3, 4]. Before the advent of modern chemotherapy and radiation therapy, arsenic was a cornerstone in leukaemia treatment [5].

In the 1970s, Chinese scientists demonstrated ATO’s efficacy against acute promyelocytic leukaemia (APL), a subtype of acute myeloid leukaemia (AML). This led to its approval by U.S. Food and Drug Administration (FDA) in 2000 (as Trisenox^®^) and European Medicines Agency (EMA) in 2017 for relapse/refractory APL [6]. Currently, ATO either alone or in combination with all-*trans* retinoic acid (ATRA), is a standard treatment for newly diagnosed and relapsed APL cases [7]. In 2010, the U.S. FDA approved the first oral formulation of ATO (Arsenol^®^), developed by researchers at the University of Hong Kong [8]. This oral formulation offers greater convenience for outpatient use, high efficacy, and significant reduction in side effects compared to intravenous administration.

Despite potent therapeutic effect, ATO exhibits significant side effects, limiting its clinical usage. To counteract the adverse off-target effects, peptide conjugates have emerged as a promising strategy for targeted delivery of ATO to leukaemia cells, enabling dose reduction while maintaining efficacy [9]. For instance, conjugation of As to peptides that have higher specificity for leukaemia and lymphoma cells, such as “tumour-homing peptides” with a sequence of CAYHRLRRC, has enabled over 1000-fold selective accumulation of a conjugate in leukaemia cell than in human blood cells, underscoring the therapeutic potential [9]. Such targeted approach not only mitigates side-effects but also holds potential for overcoming resistance in relapsed APL cases, paving the way for safer and more effective arsenic-based therapies.

Beyond ATO, several arsenic-containing compounds demonstrated significant anticancer activity [10–12], including realgar (i.e. tetraarsenic tetrasulfide (As_4_S_4_)) and various organo-arsenic compounds [5], notably, darinaparsin (ZIO-101, S-dimethylarsino-glutathione). Darinaparsin (Darvias^®^) has received Orphan Drug Designation in the United State and Europe for the treatment of peripheral T-cell lymphoma (PTCL), and was approved in Japan in June 2022 for relapsed or refractory PTCL. These advancements highlight the potentials for developing novel arsenic-based anticancer agents and expanding their therapeutic applications.

In addition to its established efficacy in APL, ATO, in combination with other agents, shows promise against various malignancies, including hematologic malignancies and other solid tumours [5, 7, 10, 13, 14]. Recent studies have demonstrated that ATO sensitizes apoptosis-resistant cancer cells, such as patient-derived colon cancer organoids, to cisplatin by targeting the ubiquitination-dependent degradation of XIAP (X-linked inhibitor of apoptosis protein), a member of the inhibitor of apoptosis proteins family. This finding suggests new therapeutic opportunities for ATO in cancer treatment [15], enhancing its role in overcoming resistance to conventional therapies.

The rational design of more effective arsenic-based anticancer drugs necessitates a comprehensive understanding of their mechanisms of action (MoA). Proteomics has been well employed to identify proteins regulated by metallodrugs [16], however, given that metals and metallodrugs frequently bind to and disrupt the function of multiple metalloproteins and metalloenzymes, proteome-wide identification of metal or metallodrug binding proteins is a perquisite for elucidating their biological and therapeutic roles [17–23]. Metallomics and metalloproteomics have emerged as pivotal disciplines for studying metals and metalloids within cells, tissues and organs [24, 25]. Notably, metalloproteomics, when integrated with other omics-approaches has significantly advanced our understanding of MoA of metallodrug [26–30].

In this review, we first provide a concise overview of certain arsenic-based anticancer drugs, highlighting their structures and anticancer properties. We then explore strategies for identifying the arsenic proteome in cells at a proteome-wide scale using metalloproteomic approaches, such as Immobilized Metal Affinity Chromatography (IMAC), Gel Electrophoresis coupled with Inductively Coupled Plasma-Mass Spectrometry (GE-ICP-MS), biotinylated arsenical pull-down, and fluorescence-based metalloproteomics. Finally, we present the latest insights into the molecular mechanism of action of arsenic-based anticancer drugs at a system level, using ATO and darinaparsin (ZIO-101) as showcases as well as such studies at a single cell level. This review aims to stimulate further research into the molecular mechanism of arsenic-based anticancer agents from a systems perspective, which provides a foundation for developing arsenic-based therapeutics for cancer therapy.

## Arsenic-based anticancer agents

Arsenic (As), a typical semimetal of group 15 elements in the periodic table alongside nitrogen and phosphorus, exists four oxidation states: -III, 0, +III and + V. The trivalent (arsenite) and pentavalent (arsenate) states are biologically stable with As(III) being particularly thiolphilic, readily forming strong bonds with thiolate groups of cysteine residues [31, 32]. Currently, several arsenic-based anticancer agents both inorganic and organic, are under investigation for clinical use. This section describes representative arsenic-based anticancer agents (drugs), their chemical structures and anticancer properties.

### Inorganic arsenic anticancer agents

**Fig. 1:**
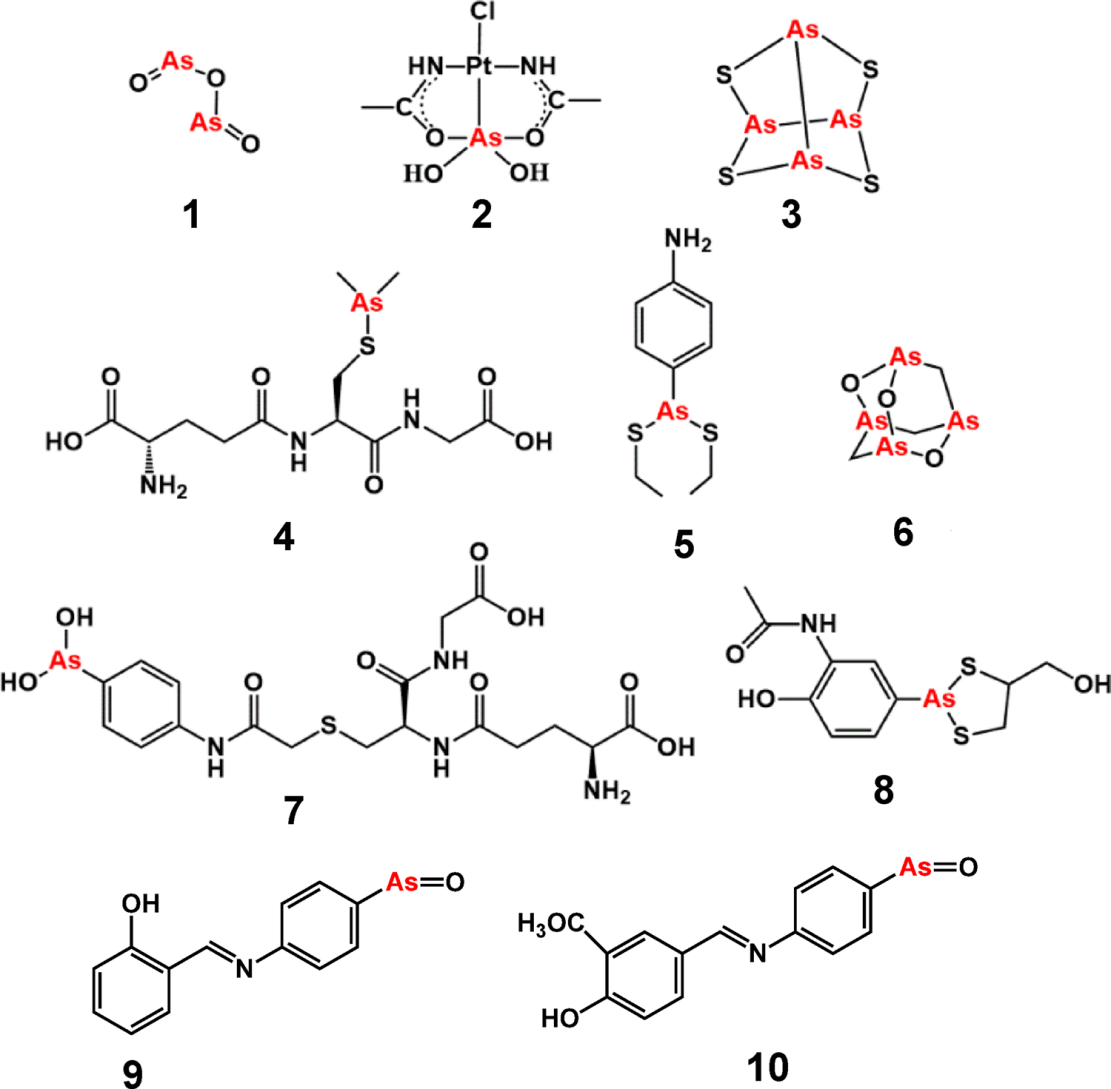
Chemical structures of representative inorganic and organic arsenic-based anticancer agents. **1**: As_2_O_3_; **2**: Arsenoplatin-1; **3**: As_4_S_4_; **4**: Darsinaparsin (ZIO-101); **5**: (2-(4-aminophenyl)-1,3,2-dithiarsinane) (PAO-PDT, 4); **6**: Arsenicin A; **7**: 4-(N-(S-glutathionylacetyl)amino)phenylarsenoxide (GSAO); **8**: (2-phenyl-1,3,2-dithiarsolan)methanol (Arsthinol); **9**: 2-(((4-oxoarsanyl)phenyl)imino)methyl)phenol; **10**: 2-methoxy-4(((4-oxoarsanyl)phenyl)imino)methyl)phenol

Arsenic trioxide ATO (**1**, Fig. 1) is polymeric in the solid state and exists mainly as H_3_AsO_3_ in neutral aqueous solutions. It has been used in traditional Chinese medicine for over two centuries and demonstrated a significant efficacy in treating APL and other cancers, particularly in combination therapies [10, 33]. ATO shows promise in treating multiple myeloma [34], myelodysplasia syndrome [35] and non-Hodgkin lymphoma [36]. Despite its success, ATO suffers limitations including systemic toxicity and poor pharmacokinetics due to rapid renal clearance of its metabolites [37]. To address these challenges, liposomal-encapsulation of ATO has been developed to decrease membrane permeability and enable its gradual release [38], improving its therapeutic profile.

Efforts to develop novel arsenic-based compounds has led to the synthesis of Arsenoplatin-1 (**2**, Fig. 1), a compound formed by heating cisplatin (cis-diamminedichlorpplatinum(II)), with As_2_O_3_ in 9 : 1 (v/v) acetonitrile/water mixture at 90 °C for three days [39]. Arsenoplatin-1 exhibits a superior anticancer activity compared with parent drugs in most cancer cells and holds a potential for delivering both platinum and arsenic species to hepatological and solid tumours [40].

Realgar As_4_S_4_ (**3**, Fig. 1), another arsenic-based compound, has been used traditionally in Chinese medicine and as a pigment. Realgar induces differentiation and apoptosis in APL cells, but is limited by low solubility and bioavailability. Realgar nanoparticles, with an average size below 100 nm, exhibit enhanced anticancer activity in melanoma cells and tumour-bearing mice with reduced toxicity [41]. Oral administration of crystalline realgar has achieved complete remission in both newly diagnosed and relapsed APL patients, indicating that As_4_S_4_ is highly effective and safe for remission induction and maintenance therapy in APL, regardless of disease stage [42].

### Organic Arsenic anticancer agents

In addition to forming stable bonds with thiolates, arsenic readily forms bonds with carbon, yielding various organo-arsenical compounds. Organo-arsenicals are generally more stable and excreted more rapidly than inorganic arsenicals, resulting in reduced toxicity. This section summarizes the structures and anticancer properties of representative organo-arsenicals.

Darinaparsin (ZIO-101, S-dimethylarsino-glutathione, Darvias^®^, **4**, Fig. 1) is a conjugate of glutathione and dimethylarsinous acid. It is one of the metabolites of inorganic arsenic during its biotransformation in the body and is a gamma-glutamyltransferase (γGT) activated prodrug [43, 44]. In tumour cells overexpressing γGT, β-elimination triggered by γGT releases the active drug from its conjugated form [45]. It exhibits superior anticancer activity compared to ATO against a range of solid tumours and hematologic malignancies [46]. Unlike ATO, darinaparsin disrupts mitochondrial membrane potential, promotes cell cycle arrest at the G2-M phase, and induces apoptosis without triggering differentiation in APL cells [47], Consequently, darinaparsin demonstrates selectivity and efficacy in ATO-resistant cell lines. It has received orphan drug designation in the United State and Europe for the treatment of peripheral T-cell lymphoma (PTCL) and its intravenous formulation was approved in Japan in June 2022 for relapsed or refractory PTCL [44].

GSAO (4-(*N*-(*S*-glutathionylacetyl)amino) phenylarsenoxide) (**7**, Fig. 1) is another glutathione conjugate with phenylarsonous acid that targets cancer cells by disrupting mitochondrial function through inhibition of adenine nucleotide translocase (ANT). Like ZIO-101, GSAO is activated by γGT in tumor cells, where its arsenical moiety could bind to cysteine residues of ANT, impairing its function, and leading to cell proliferation arrest and cell death [48]. GSAO demonstrates efficacy against various cancers, including pancreatic, lung, prostate and fibrosarcoma tumors in mice, with minimal side effects [48]. It is currently in Phase I clinical trials for patients with advanced solid tumors unresponsive to standard therapies [49] (https://clinicaltrials.gov/study/NCT01147029).

Moreover, several organo-arsenic compounds (Fig. 1) have been synthesized and evaluated for antitumor activity across various cancer cell lines. Two oxoarsanyl compounds (**9**, **10**), 2-(((4-(oxoarsanyl)phenyl) imino)methyl)phenol (C_13_H_10_AsNO_2_) and 2-methoxy-4-(((4-(oxoarsanyl)phenyl)imino)methyl) phenol (C_14_H_12_AsNO_3_), exhibit potent growth inhibition of HL-60 cells, with IC_50_ values of 0.77 and 0.51 µM, respectively. Both compounds induced apoptosis via oxidative stress in HL-60 cells [50].

Several dithiaarsenes also show promising anticancer activity. For example, (2-(4-aminophenyl)-1,3,2-dithiarsinane, **5**, Fig. 1) inhibits HL60 growth with IC_50_ values of 0.7 and 0.6 µM after 48 and 72 h of treatment, respectively. This activity is attributable to its binding to the C-terminal cysteine pair of thioredoxin reductase (TrxR), inducing oxidative stress-mediated apoptosis in HL-60 cells [51].

Arsthinol ((2-phenyl- [1–3]dithiarsolan-4-yl)-methanol, **8**, Fig. 1), an antiprotozoal compound synthesized in 1949 [52], exhibits antitumor activity against leukemia cell lines with a superior therapeutic index compared to ATO [53]. Its efficacy highlights its potential as an anticancer agent.

Arsenicin A (2,4,6-trioxa-1,3,5,7-tetrarsatricyclo [3.3.1.13,7] decane, **6**, Fig. 1) with a formulation of C_3_H_6_As_4_O_3_, is a member of polyarsenical nature product isolated from the marine sponge *Echinochalina bargibanti*. It demonstrates a 21-fold greater anti-proliferative activity than ATO in NB4 cells [54]. Several arsenicin A analogues have been synthesized and evaluated against various cancer cell lines, showing enhanced cytotoxicity compared to ATO in solid tumor cell lines, including colon, melanoma, ovarian cancer, renal cancer, prostate cancer and breast cancer [55]. These findings highlight the therapeutic potential of polyarsenicals and warrant further investigation for anticancer applications.

## Systemic identification of arsenic-binding proteins by metalloproteomics

Trivalent arsenic (As(III)), known for its thiolphilic properties, binds to numerous proteins and enzymes, influencing the therapeutic and carcinogenic effects of arsenic. Though interactions between ATO and individual proteins have been extensively characterized [12, 31], such studies may provide limited insights into the broader molecular landscape. Systemic approaches, including transcriptomic, chemogenomic and proteomics, have been employed to elucidate the anticancer mechanisms of ATO [56, 57]. However, these primarily capture cellular response rather than identifying direct arsenic-binding proteins. Proteome-wide identification of these proteins in cells or organisms is essential for a comprehensive understanding of the molecular mechanism of action of arsenic-based drugs from a system perspective. Advancements in analytical techniques have enabled the identification of arsenic-binding proteins in living cells [58]. Previously, metalloproteomic approaches have successfully identified metal-binding proteins in various cells at a proteome-wide scale [17, 20, 22, 29, 30, 59–62], and similar metalloproteomic approaches have been adapted to characterize the arsenic proteome at a systems level (vide infra). This review summarizes these metalloproteomic methods and their application to arsenic-binding protein identification.

### Immobilized Metal Affinity Chromatography

Immobilized Metal Affinity Chromatography (IMAC) is a robust technique for isolating and purifying proteins based on their interactions with metal ions and/or metallodrugs immobilized on a solid support. When coupled with other techniques like protein identification techniques, such as matrix-assisted laser desorption/ionization mass spectrometry (MALDI-MS), IMAC enables the enrichment and identification of metal-binding proteins at a proteome-wide scale. This approach is particularly valuable for characterization of metal-binding proteins or binding motifs relevant to cellular functional behaviors of metals [62]. Similar to other IMAC implementations, arsenic affinity chromatography employs arsenic or arsenical-containing molecules immobilized on a matrix via a linker. When biological samples pass through the column, arsenic-binding proteins are retained due to the interactions between cysteine thiol groups and immobilized trivalent arsenicals, the arsenic bound proteins are then eluted using a buffer containing a strong reducing thiol such as dithiothreitol (DTT) or β-mercaptoethanol (β-ME, 2-mercaptoethanol). These thiols competitively displace the bound proteins from the immobilized trivalent arsenicals by breaking the As(III)-thiolate bonds formed with the cysteine residues. The first arsenic-based IMAC utilized *p*-aminophenylarsine oxide (PAPAO) covalently linked to CNBr-activated Sepharose 6B to separate proteins containing mono- and dithiols [63]. Phenylarsine oxide (PAO), with at least two thiol-binding sites, forms stable dithioarsenic complex with proteins containing vicinal thiols. For instance, using a PAO-agarose affinity column after extensive washing with buffers containing either 20 mM β-ME or DTT, three differentially expressed proteins, e.g., galectin 1 was identified in the β-ME-eluted fraction from Chinese hamster ovary (CHO) cells; while glutathione-S-transferase P-form and thioredoxin peroxidase II in the β-ME- and DTT-eluted fractions from an arsenic-resistant CHO cells. The binding of galectin 1 and thioredoxin peroxidase II with As(III) was further validated by co-immunoprecipitation [64].

**Fig. 2:**
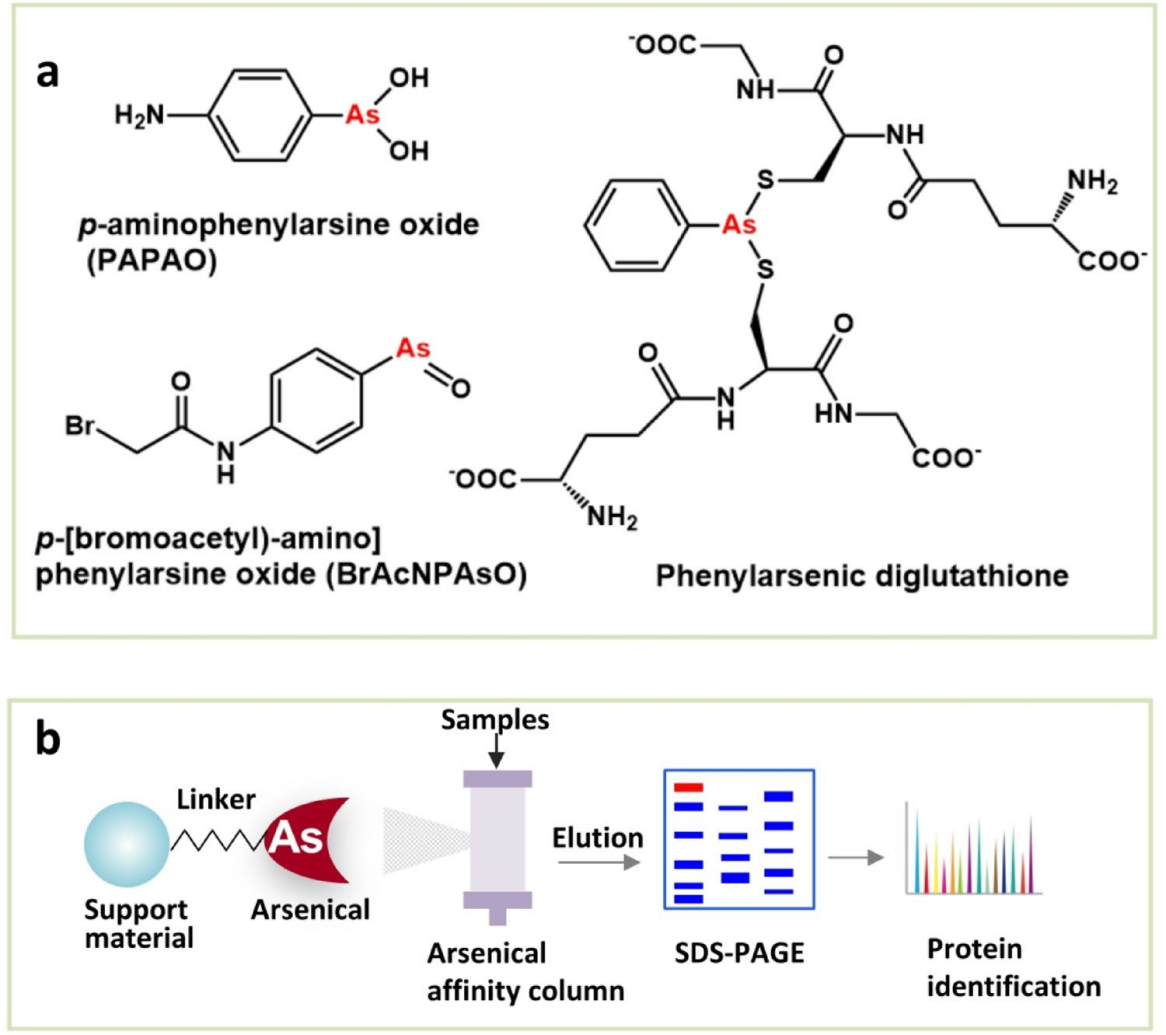
(**a**) Chemical structures of representative arsenic-binding moieties used in the resins. (**b**) General scheme showing arsenic-binding protein identification by arsenic-based affinity choromatography coupled with gel electrophoresis and mass spectrometry

A challenge in arsenic affinity chromatography is the oxidation of thiol groups which can abolish arsenic binding. The reducing agent tris(2-carboxyethyl)-phosphine (TCEP) effectively maintains thiols in their reduced a state, facilitating binding to PAO. For instance, TCEP enabled PAO affinity chromatography to identify albumin and triose phosphate isomerase as arsenic-binding proteins from a vicinal thiol-containing proteins in a detergent-soluble rat brain extract, offering a strategy for discovering lower abundance, redox-active regulatory proteins [65]. Subsequently, various arsenic-binding resins have been developed, incorporating functional groups such as *p*-aminophenylarsine oxide (PAPAO), *p*-[(bromoacetyl)-amino]phenylarsenoxide (BrAcNPAsO), and phenylarsenic diglutathione [31, 66, 67] and their chemical structures are shown in Fig. 2a. Additionally, the biarsenical fluorescent probe, FlAsH, originally developed for in vivo imaging and purification of proteins with genetically fused tetra-cysteine tags [68, 69], has been adapted as a resin to identify endogenous arsenic binding proteins in cells [70]. These methods are particularly effective for isolating proteins with multiple or adjacent cysteines residues, enhancing the study of arsenic-protein interactions.

Coupling arsenic affinity chromatography with gel electrophoresis and mass spectrometry enabled proteome-wide identification of arsenic binding proteins in biological samples [71, 72] (Fig. 2b). Using affinity resins prepared by reacting Eupergit C with *p*-aminophenylarsine oxide, arsenic-bound proteins were purified via arsenic affinity chromatography eluted with DTT-containing buffer with or without 1% SDS, and subjected to in-gel or in solution digestion before LC-ESI-MS/MS analysis. This approach allowed 50 arsenic-binding proteins in the nuclear fraction and 24 in the membrane/organelle fraction in A549 human lung carcinoma cells, to be identified [71].

Systemic identification of arsenic-binding proteins were also carried out using a PAO-based affinity chromatography combined with two-dimensional electrophoresis (2-DE) for protein separate, followed by MALDI-Q-TOF-MS for identification [72]. This method identified 19 arsenic-binding proteins in CHOA and SA7 cells, primarily enriched in metabolic, stress and developmental process as determined by GO enrichment analysis. Sequence analysis revealed that reticulocalbin-3 precursor (RCN3) lacks cysteine residues, while heat shock protein 27 (HSP27) and peroxiredoxin 6 (Prdx6) have only one cysteine residue. Notably, recombinant HSP27 and RCN3 bound to PAO-agarose matrix, whereas Prdx6 did not, indicative of cysteine residues being not indispensable for arsenic binding [72].

Additionally, arsenic-binding proteins can be purified using gel filtration and anion exchange column chromatography, followed by SDS-PAGE and identification via MALDI-MS. This approach enabled a ternary dimethylarsinous-hemoglobin-haptoglobin complex as the major arsenic-binding protein in rat plasma [73].

### GE-ICP-MS

**Fig. 3:**
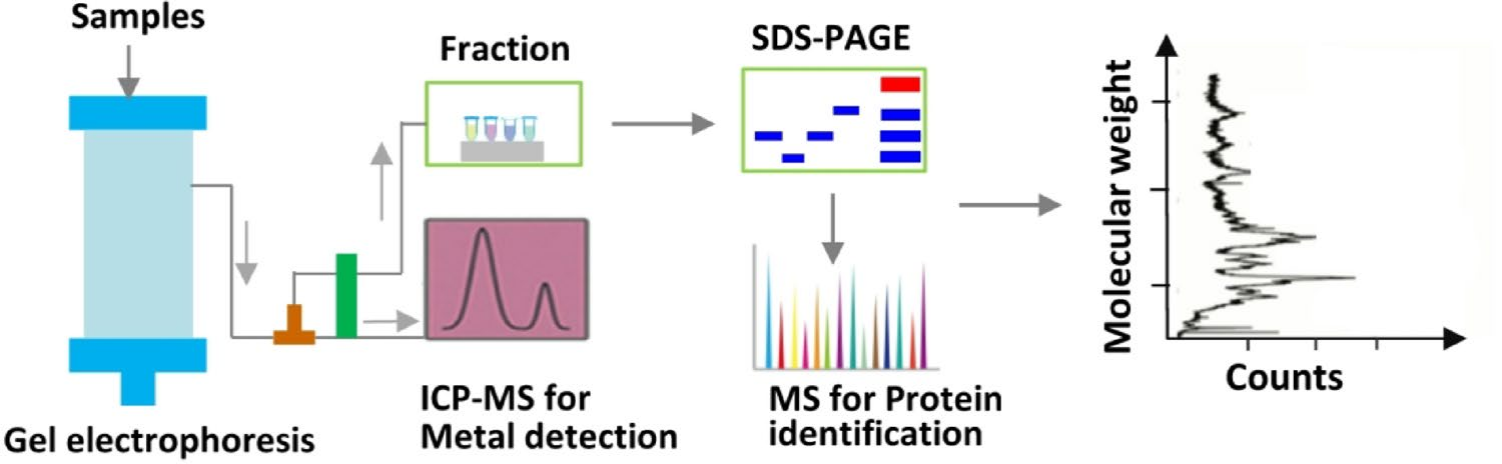
Shcematic workflow of GE-ICP-MS shows simultaneous detection of metal by ICP-MS and protein identification by MALDI-TOF-MS, allowing metal-associated proteins to be identified

A novel metalloproteomics approach, continuous-flow electrophoresis coupled with inductively coupled plasma mass spectrometry (GE-ICP-MS, Fig. 3) has been developed to identify metal-binding proteins [23, 74]. This method employs column-type gel electrophoresis, using traditional slab gel preparations with compositions tailored to the target proteins. As samples passes through the column, the eluant is split via a T-connection, enabling simultaneous metal ion detection by ICP-MS and protein identification by biological mass spectrometry. This technique has previously identified Bi^3+^-binding proteins [20, 75], and Ag^+^-binding proteins in bacterial cells treated with silver compounds and silver nanoparticles (AgNPs) [76]. Upon combination with liquid chromatography (LC-GE-ICP-MS), which enhances separation resolution, leading to identification of silver proteome in both *E. coli* [19] and *S. aureus* [77].

This method was applied to track arsenic-binding proteins in NB4 cells (APL) and HL60 (AML) cells upon treatment with ATO or darinaparsin [78]. Notably, histone H3.3 was found to be the only arsenic-binding protein in both cell lines treated with ZIO-101, but not ATO with the binding confirmed in vitro. The failure to identify low-abundance proteins or those with weak arsenic binding may result from dissociation during gel electrophoresis. These findings suggest a distinct mechanism of action for ATO and ZIO-101. Comprehensive identification of arsenic-binding proteins remains essential to fully elucidate the molecular mechanisms of action of arsenic-based anticancer drugs.

### Biotinylated Arsenical Pull-down

An arsenic-biotin conjugate has been developed to capture arsenic-binding proteins [79]. For instance, Chen’s group synthesized an arsenic-biotin conjugate by coupling the penta-fluorophenol ester of biotin with *p*-aminophenylarsenoxide. The MCF-7 cells treated with this conjugate were subjected to streptavidin resin to isolate arsenic-binding proteins, which were then separated by denaturing gel electrophoresis, in-gel digested, and identified *via* mass spectrometry (i.e. MALDI-MS). Fifty arsenic-binding proteins in MCF-7 cells were identified [79]. Two proteins, β-tubulin and pyruvate kinase M2 (PKM2), were confirmed as arsenic-binding proteins in vitro. Arsenic binding to β-tubulin inhibits tubulin polymerization, disrupting its function, whereas binding to PKM2 does not affect its enzymatic function, likely due to cysteine residues being distant from the active site [79]. These findings highlight the importance of distinguishing arsenic-binding proteins to identify therapeutic targets in cancer cells.

Using a similar approach, promyelocytic leukemia protein (PML) and PML-retinoic acid receptor alpha (PML-RARα) were identified as arsenic-binding proteins in APL cells [80, 81]. In another study [82], APL cells treated with biotinylated arsenic were processed using streptavidin pull-down, followed by liquid chromatography-tandem mass spectrometer (LC-MS/MS) analysis, identifying over 40 arsenic-binding proteins. Heat shock protein 9 (HSPA9 with molecular mass of 70 kDa), glutathione S-transferase P1 (GSTP1) and pyruvate kinase M2 (PKM2) were validated in vitro for arsenic binding [82], providing a foundation for investigation pathways modulated by ATO.

Additionally, globally profiling of arsenic-binding proteins was achieved using a human proteome microarray consisting of 16,368 affinity-purified N-terminally GST-tagged proteins [83]. Biotinylated *p*-aminophenylarsenoxide was incubated with the microarray and arsenic-binding proteins were detected using Cy3-conjugated streptavidin, with green florescence indicating arsenic-binding proteins. This approach identified 360 arsenic-binding proteins, offering a valuable resource for elucidating the mechanism of action of ATO in cancer cells [83].

**Fig. 4:**
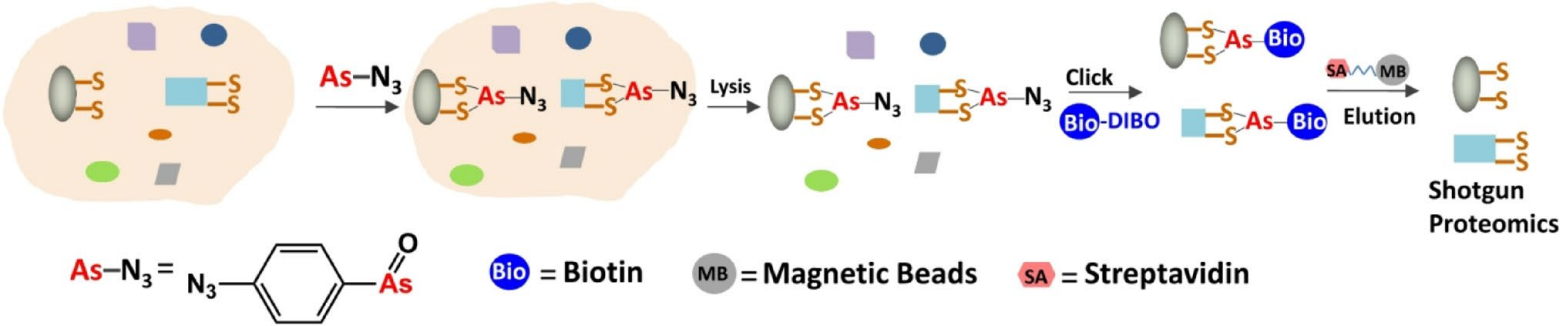
Identification of arsenic-binding proteins via *p*-azidophenylarsenoxide (PAzPAO)-based metalloproteomics. PAzPAO binds to cellular proteins *via* its trivalent arsenical, while its azide undergoes a “click” reaction with dibenzylcyclooctyne (biotin-DIBO), enabling the purification of arsenic-binding proteins, which can be identified by shotgun proteomics

To address the limitation of arsenic-biotin conjugates, which are bulky and may interfere with endogenous biotin pathway, another approach using *p*-azidophenylarsenoxide (PAzPAO) as a compact arsenical probe was developed to capture arsenic-binding proteins in cells [84] (Fig. 4). PAzPAO entered cells, where its trivalent arsenical moiety binds to cellular proteins, and subsequently its azide group enables formation of a biotin-dibenzylcyclooctyne(DIBO)-protein conjugate via click chemistry, facilitating efficient labelling of arsenic-binding proteins. These proteins are then purified using streptavidin-coated magnetic beads [84]. Coupled with shotgun proteomics, this method identified 48 arsenic-binding proteins in A549 human lung carcinoma cells, including antioxidant proteins and glyceraldehyde-3-phosphate dehydrogenase [84]. This approach provides a valuable strategy for examining the roles of arsenic in health effects and cancer therapy.

### Fluorescence-based metalloproteomics

Fluorescence-based metalloproteomics has been employed for proteome-wide identification of metal-binding proteins [23, 85], including those binding Bi^3+^ [20, 22, 86], Cu^2+^, Zn^2+^, Ni^2+^ [87], Fe^3+^ [88], Ga^3+^ [89] and Cr^3+^ [60] in various cell types, facilitating studies on the biological and therapeutic roles of metals and metallodrugs. This approach utilizes membrane permeable fluorescence probes, such as M^*n+*^*-TRACER*, which consists of a metal-nitrilotriacetate (NTA) moiety, a fluorophore and an arylazide. Guided by the metal-NTA moiety, these probes bind to intracellular proteins, including those with genetically fused His_6_-tag, exhibiting significant fluorescence turn-on [90]. Upon UV activation, the arylazide anchors the probes to the proteins of interest, enabling downstream protein identification by proteomic approach [23]. This strategy is also applicable for tracking arsenic-binding proteins in living cells, provide appropriate arsenical probes are developed.

Given that vicinal dithiol proteins (VDPs), characterized by at least one pair of vicinal closely spaced thiols, are key arsenic-binding proteins, fluorescent probes designed to selectively image and track endogenous VDPs can be adapted for proteome-wide identification of arsenic-binding proteins. VDPs are targeted by fluorescent As(III)-based probes through a dynamic thiol exchange process that leverages the high affinity of trivalent arsenic for closely spaced cysteine thiols. This process is essential for both selective binding to the target protein and activation of the probe’s fluorescence, typically via a turn-on mechanism. Such a thiol exchange mechanism enables proteome-wide profiling by enriching arsenic-bound proteins for mass spectrometry or imaging, with specificity enhanced by the probe’s design to minimize off-target monothiol interactions.

**Fig. 5:**
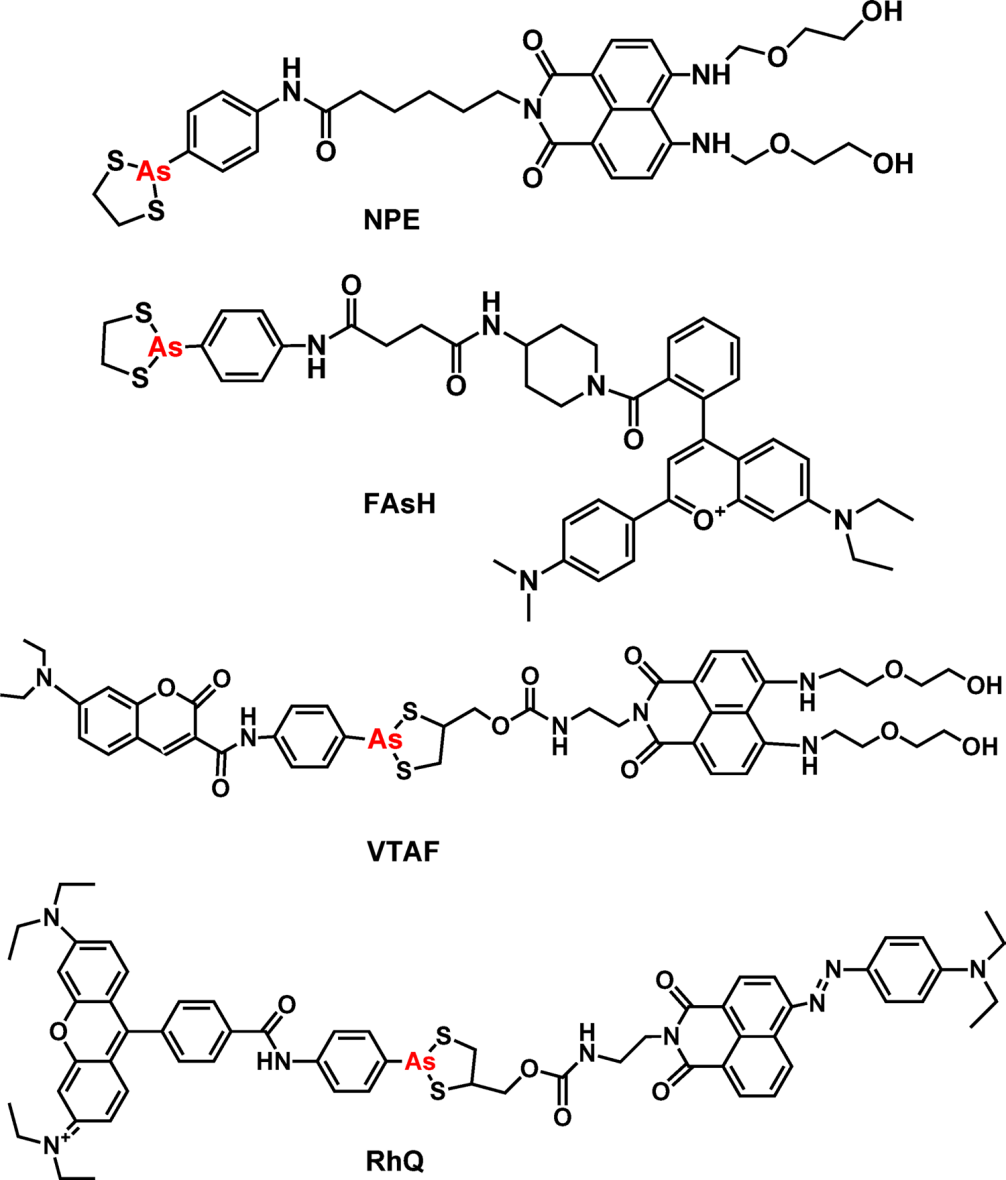
Chemical structures of representative monoarsenical fluorescent probes

By conjugation of arsenicals with a fluorophore *via* a linker, several monoarsenical fluorescent probes have been developed, including NPE, FAsH, VTAF, RhQ (Fig. 5). A comprehensive review of VDP visualization using monoarsenical probe is available elsewhere [91]; here, selected probes are highlighted as examples. Utilizing naphthalimide as the fluorophore and cyclic dithiaarsanes as the arsenic-binding group, a highly selective and cell-permeable fluorescent probe (NPE) was synthesized, which could rapidly detect and image VDPs both in vitro and in living cells [92]. The approach allowed non-invasive studies of redox-regulated signaling pathways involving VDPs. To minimize fluorescence background from unreacted or non-specifically bound probes, an “off-on” fluorescent probe (FAsH) was developed by employing a flavylium dye as the fluorophore and a dithiarsenolane group as the arsenic-binding motif. FAsH selectively targets VDPs in cells, exhibiting a significant fluorescence turn on due to hydrophobic interaction and restricted intramolecular rotation upon entering hydrophobic domains [93]. In order to quantify global distribution and dynamic change of VDPs in living cells, a ratiometric fluorescent probe (VTAF) was designed based on **f**luorescence **r**esonance **e**nergy **t**ransfer (FRET) [94]. VTAF uses a coumarin and a naphthalimide for fluorophores for signal transduction and aminophenyl-1,3,2-dithiarsenolane as arsenic-binding motif. This probe enables self-calibration fluorescence signals, allowing quantification of the global distribution and dynamic changes of endogenous VDPs in living cells. VTAF represents the first ratiometric fluorescent probe for highly selective detection and visualization of subcellular VDP distribution [94].

All VDPs proteins labelled by arsenical fluorescent probes can be separated by electrophoresis such as two-dimensional electrophoresis (2-DE), and identified using mass spectrometry techniques, such as matrix-assisted laser desorption/ionization time-of-flight mass spectrometry (MALDI-TOF-MS). A recent study introduced an “off-on” fluorescent probe (i.e. RhQ) [95], designed with rhodamine as the fluorophore and naphthalimide as the quencher, and a trivalent arsenic moiety as the linker. Initially quenched by the naphthalmide-azo conjugate, RhQ’s rhodamine fluorescence is restored upon interaction with VDPs, due to the disruption of FRET. This enables RhQ to image VDPs in living cells with minimal background fluorescence or interference from subcellular environmental changes. Using RhQ, dynamic tracing of VDPs on the surface of live MCF-7 cells was achieved. By combining fluorescent imaging with proteomics, several VDPs were identified, including thioredoxin reductase 1 (TrxR1), protein disulfide-isomerase (PDI), medium-chain specific acyl-CoA dehydrogenase and malate dehydrogenase (MDH) [95]. However, a limitation of such probes is potential dissociation of the arsenic probe from bound proteins during electrophoresis, reducing the number of identified proteins.

**Fig. 6:**
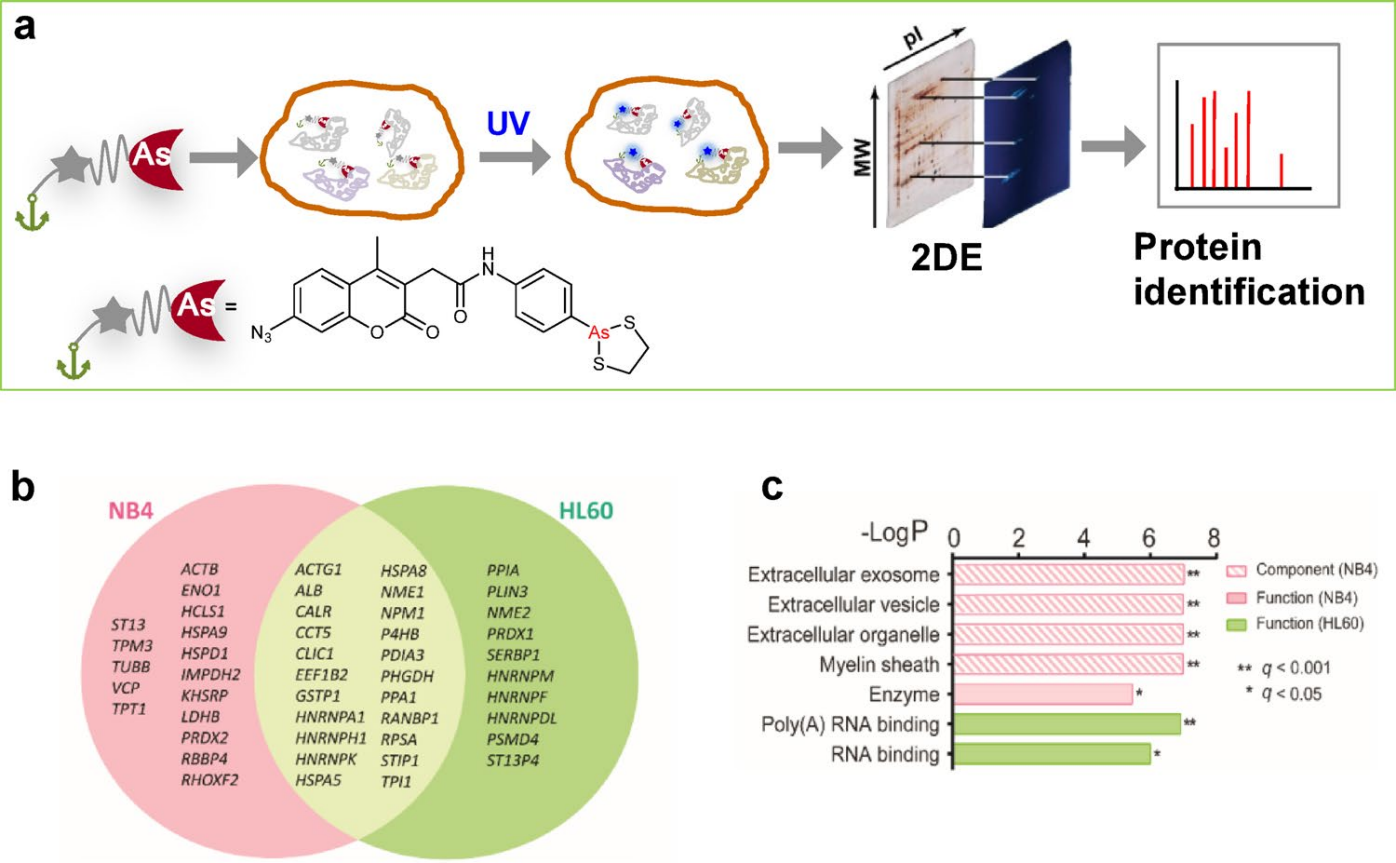
(**a**) Schematic representation of fluorescence-based metalloproteomic approach via a unique organoarsenic probe for identifying arsenic-binding proteins in live cells. (**b**) Summary of the identified arsenic-binding proteins by As-AC probe in NB4 and HL60 cell lines with 22 common proteins. (**c**) GO enrichment analysis of NB4- and HL-60-specific arsenic-binding proteins. The p-value was set as ‘< 0.001’, q-value ‘< 0.05’ in functional categories and ‘< 0.001’ in component categories are listed. The Fig. 6b and c were from reproduced with permission from ref27. Copyright 2021 the Royal Society of Chemistry

To address this, a photo-affinity organoarsenic probe, As-AC was developed by conjugating a trivalent arsenic moiety with a coumarin fluorophore and an arylazide [30]. As-AC rapidly enters cells to label intracellular arsenic-binding proteins specifically via binding to cysteine residues, resulting in an eight-fold fluorescence enhancement. Upon UV activation, the arylazide forms covalent bonds with labelled proteins, anchoring the probe and enabling downstream protein identification *vi*a high-throughput proteomics (Fig. 6a). This covalent attachement ensures retention of fluorescene in the gel even if arsenic dissociates from weakly or transiently bound proteins, unlike non-photo-activated probes where dissociation occurs during electrophoresis. Using As-AC, approximately 38 and 32 arsenic-binding proteins were identified in NB4 and HL60 cells, respectively, with 22 proteins common to both cells (Fig. 6b). Gene Ontology (GO) enrichment analysis revealed that NB4-specofic proteins are primarily associated with enzymatic functions and enriched in exosome and myelin sheath, whereas HL60-specific proteins exhibit RNA-binding properties without specific localization [30] (Fig. 6c). These differences may explain the distinct responses of the two cells against ATO.

### Molecular mechanim of action of arsenic trioxide from a system perspectives

**Fig. 7:**
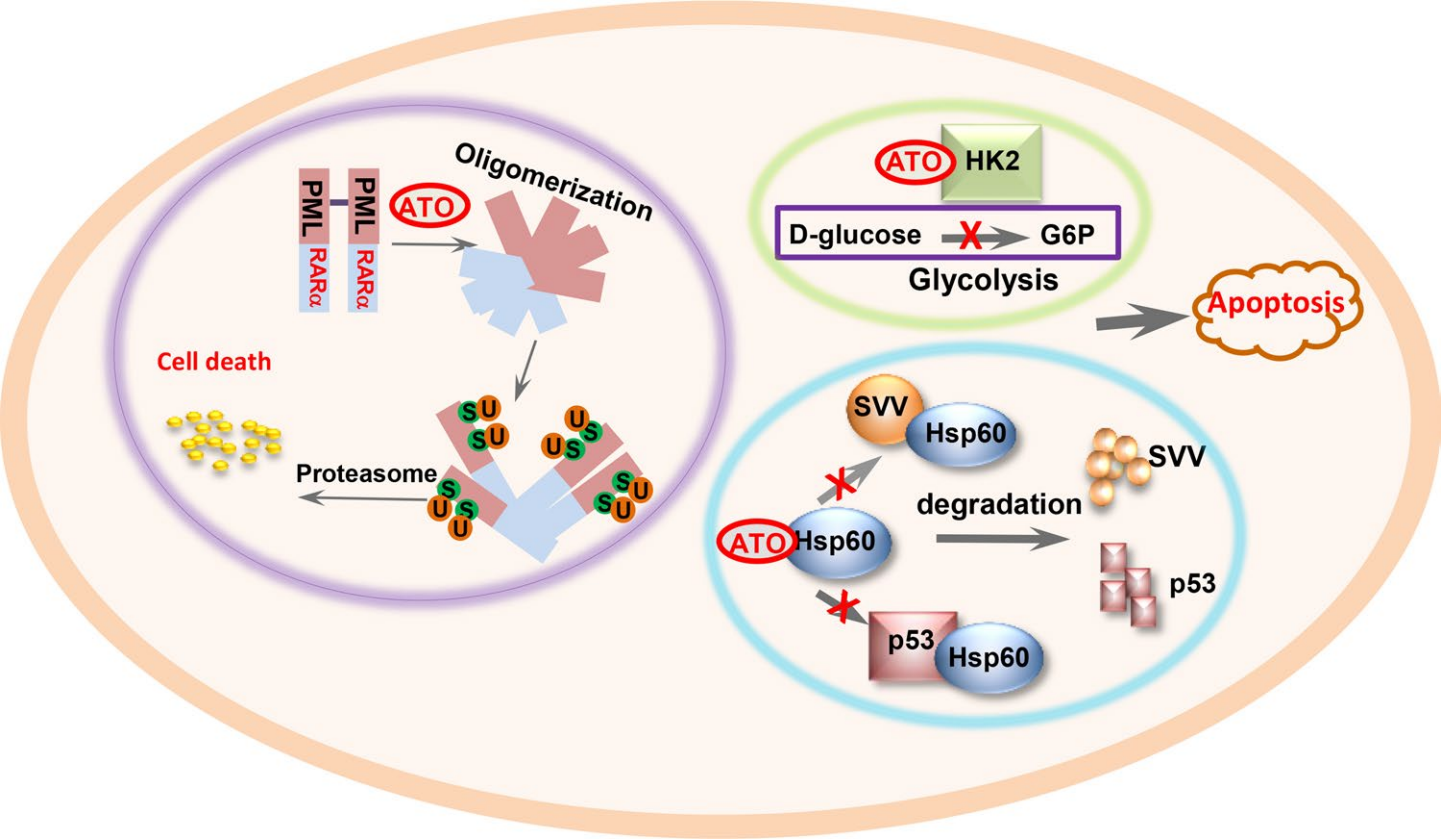
Proposed mechanism of arsenic trioxide against cancer cells. ATO binds to an oncogenic fusion protein PML-RARα, resulting in enhanced SUMOylation, ubiquitination and subsequent degradation, and eventually cancer cell death. ATO also disrupts gycolysis in cancer cell through binding and funcitonal disruption of HK2. Additionally, ATO binds to Hsp60, abolishing its ability to recruit partner proteins, survivin and p53, leading to their degrdation, and subsequent cancer cell death through apoptosis. PML: promyelocytic leukemia protein; RARα, retinoic acid receptor alpha; HK2: hexokinase-2; SVV; survivin; G6P: glucose-6-phosphate

Enormous research has elucidated the machanism of action of ATO in treating APL. ATO induces cancer cell differentiation or apoptosis, depending on dose, cell type and exposure duration [7, 96]. Early studies revealed that ATO promotes degradation of the oncogenic PML-RARα fusion proteins by directly binding to cysteines in the zinc fingers of the N-terminal RING domain of PML and PML-RARα, resulting in oligomerization, leading to enhanced SUMOylation, ubiquitination and subsequent degradation (Fig. 7) [7, 97, 98]. However, ATO’s efficacy in systems lacking PML-RARα and its anticancer activity in non-APL cancer suggest addtional biological targets [99]. Given arsenic’s thiophilic nature and its ability to bind to numerous proteins [31], identifying key molecular targets of ATO remains challenging. Although several targets, including peptidyl-prolyl cis-trans isomerase NIMA-interacting 1 (PIN1) [100], methlentetrahydrofolate dehydrogenase 1 (MTHFD1) and mutated Nucleophosmin-1 (NPM1) [101, 102] have been identified, proteome-wide profiling of arsenic-binding proteins offers critical insights into ATO’s mechanisms of action from a system perspective.

Global profiling using a human proteome microarray containing 16,368 proteins identified 360 candidate arsenic-binding proteins [83]. Bioinformatic analysis revealed significant enrichment in glycolysis amongst 16 Gene Ontology (GO) terms (*P* < 0.01) with many identified proteins being glycolytic enzymes. Amongst the proteins identified as glycolytic enzymes, notably, hexokinase-1 (HK1) was confirmed as an arsenic-binding protein. Although HK2, a homologous isoform prevalent in insulin-sensitive tissues and highly expressed in tumors with enhanced aerobic glycolysis [103, 104] was absence from the microarray, its arsenic binding was validated using a biotinylated arsenic probe (Biotin-As) and CY3-strpatavidin (Cy3-SA), with a binding affinity *K*_d_ of 12.3 nM. Cys256 and Cys704 were identified as critical residues for this interaction [83]. Metabolomics analysis in the SGC7901 gastric cancer cell line confirmed arsenic binding to HK2 significantly inhibited glycolysis. Furthermore, HK2 overexpression in cancer cells mitigated arsenic-induced apoptosis [83], suggesting that glycolysis, particularly HK2, is a key target of ATO in cancer cell death (Fig. 7). However, as these arsenic-binding proteins were identified using a proteome microarray rather than directly from cancer cells, the findings may not fully reflect the in vivo context, necessitating further validation in cellular systems.

Recent studies have intergrated metalloproteomics with proteomics to directly track arsenic-binding proteins in cancer cells. In NB4 and HL60, 38 and 32 arsenic-binding proteins were identified respectively, with 22 common to both cell lines, suggesting these shared proteins are unlikely to be specific targets of arsenic trioxide. Proteomics analysis of NB4 cells treated with ATO revealed 250 differentially expressed proteins, including 141 up-regulated and 109 down-regulated proteins. Bioinformatic analysis of unique arsenic-binding proteins in NB4 cells combined with arsenic-regulated proteins indicated involvement in diverse biological processes, including translational initiation, viral transcription, protein folding, endoplasmic reticulum (ER) translocation, nonsense-mediated mRNA decay, rRNA processing, RNA binding, protein binding and unfolded protein binding. These findings suggest that ATO induces broad functional disruption in NB4.

A protein-protein interaciton (PPI) network of arsenic-associated proteins in NB4 cells identified the top 10 proteins with the highest betweenness centrality scores : HSPD1, HSPA5, HSPA8, HSPA9, LAMP1, HNRNPA1, CASP3, RPS6, ACTB and CCT5. Among them, HSPD1 (Hsp60), the most up-regulated arsenic-binding protein, has been previously exploited as a potential target for cancer therapies [105, 106]. Consequently, Hsp60 was investigated as a novel ATO target. Further study confirmed that ATO binds to Hsp60 via cysteines residues, and such a binding was also validated in cellular systems. This binding attentuated Hsp60’s ATPase activity and abolished its protein refolding activity. Moreover, arsenic binding disrupts its abilities to recruit proteins such as p53 and survivin, leading to their degradation via the proteasomal pathway (Fig. 7). This study identifies Hsp60 as a key ATO target in cancer cells, and establishes a robust platform for system-wide analysis of the mode of action of metallo-anticancer drugs. Given that several hub proteins have been identified, further investigation of these proteins may provide deeper insights into the molecular mechanism of action of ATO from a systems perspective.

### Molecular mechanism of action of ZIO-101 at a system perspective

The molecular mechanism of ZIO-101 (darinaparsin) has been comprehensively studied and reviewed [107–109], highlghting its distinct actions compared to ATO. ZIO-101 induces mitochondrial disruption, reactive oxygen species (ROS) production, cell cycle arrest, apoptosis and modulation of oncogenic signaling pathways. However, a comprehensive system-wide understanding of its anticancer mechanims remains unclear. While transcriptomics has been widely used to identify gene expression changes, pathways and unique responses to ZIO-101 [110, 111], proteome-wide identification of ZIO-101 binding proteins offers direct insights into the biological processes underlying its anticancer activity.

**Fig. 8:**
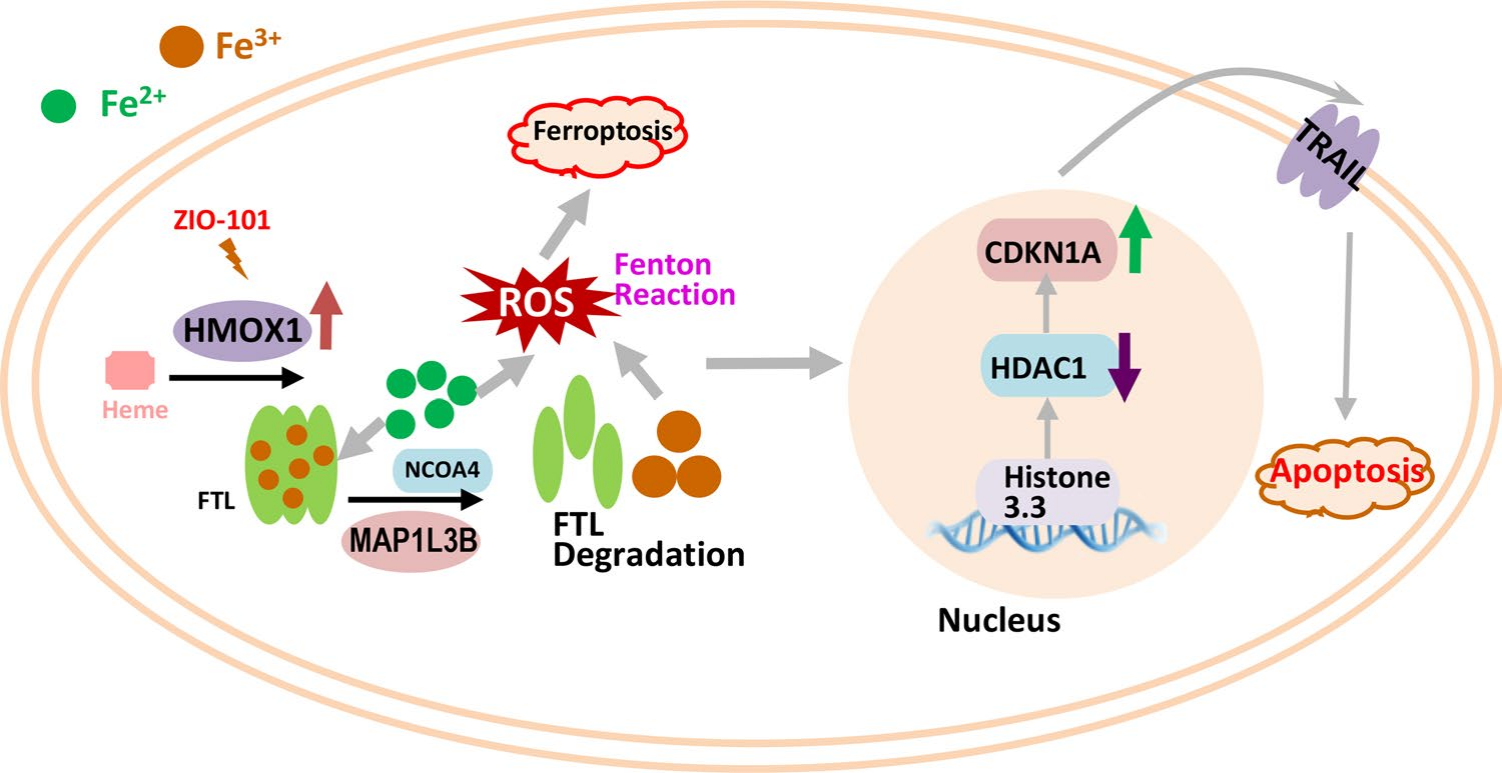
Proposed mechanism of ZIO-101 against cancer cells from a systems perspective. ZIO-101 upregulates HMOX1 expression, as well as various ferroptotic genes, degradating FTL, with excessive iron release, finally triggering ferroptosis. Subsequently, ZIO-101 enters into nucleus and binds to Histone3.3, destabilizing nucleosomes, further regulating HDAC1, and CDKN1A, eventually promoting TRAIL-induced apoptosis

Metallopoteomics using GE-ICP-MS has been utilized to identify both ATO and ZIO-101 binding proteins in leukemia cells including NB4 cells (APL) and HL60 cells (acute myeloid leukemia (AML)) [78]. Notably, histone H3.3 was identified as a ZIO-101-binding protein, but not an ATO-binding protein, implicating distinct molecular mechanisms. Binding of ZIO-101 to H3.3 mediated by Cys111, was validated both *in cellulo* and in vitro. This interaction disrupts histone H3.3 dimerization, destabilizing nucleosomes, downregulating histone deacetylase 1 (HDAC1), and significantly upregulating cyclin-dependent kinase inhibitor 1 A (CDKN1A). This cascade promotes TRAIL-induced apoptosis, a programmed cell death pathway (Fig. 8). Knockdown of histone H3.3 attenuatd ZIO-101 induced upregulation of apoptosis-related genes, confirming H3.3 as a critical ZIO-101 target [78].

Gene expression profiling revealed distinct protective signaling responses between darinaparsin and ATO. In myeloma cell lines (U266 and 8226/S), ZIO-101 (but not ATO) markedly increased expression of the pro-apoptotic gene *Noxa*, indicating a unique mechanism that may render ZIO-101 effective against ATO-resistant multiple myeloma [110].

Genome-wide transcriptional analysis in NB4 cells further elucidated the underlying mechanism of ZIO-101 [111]. Bioinformatic analysis of differenitally expressed genes (DEG) revealed that ferroptosis was the most activated pathway at 6 and 12 h post ZIO-101 treatment, shifting to apoptosis induction at 24 h. This suggests ZIO-101 triggers ferriptosis at the early stage, and apoptosis at later stage, in consistence with a previous report [107]. Biophysical studies confirmed ZIO-101-induced iron accumulation and lipid ROS production. Genes invovling in iron homeostasis, particularly HMOX1 (heme oxygenase 1), were significantly upregulated. Additionally, the upregulated genes relevent to iron homeostasis also include gene ecoding ferritin light chain (FTL) protein, the gene encoding microtubule-associated proteins 1 A/1B light chain 3B (MAP1LC3B) and the gene encoding nuclear receptor coactivator (NCOA4). Silencing HMOX1 effectively attenuated ZIO-101-induced ferroptosis, with significantly alleviated iron accumulation and lipid peroxidation. This study demonstrates that ZIO-101 significantly stimulates HMOX1 overespression, enhancing iron accumulation and upregulating ferroptotic genes FTL, MAP1LC3B and NCOA4. Subsequently, FTL is degradated, resulting in iron release, triggering Fenton reaction, lipid oxidation and ferroptosis eventually (Fig. 8).

### Single cell analysis of arsenic-based anticancer drugs

Most studies on the cytotoxicity and mechanims of arsenic-based drugs or compounds have been conducted in bulk systems. However, single-cell analysis offers greater precision in elucidating intrinsic mechanisms by capturing cell-to-cell heterogeneity in size, protein levels and RNA transcript expression [112]. This approach provides a more accurate representation of celluar variation, enhancing our understanding of fundamental biological principles [113].

**Fig. 9:**
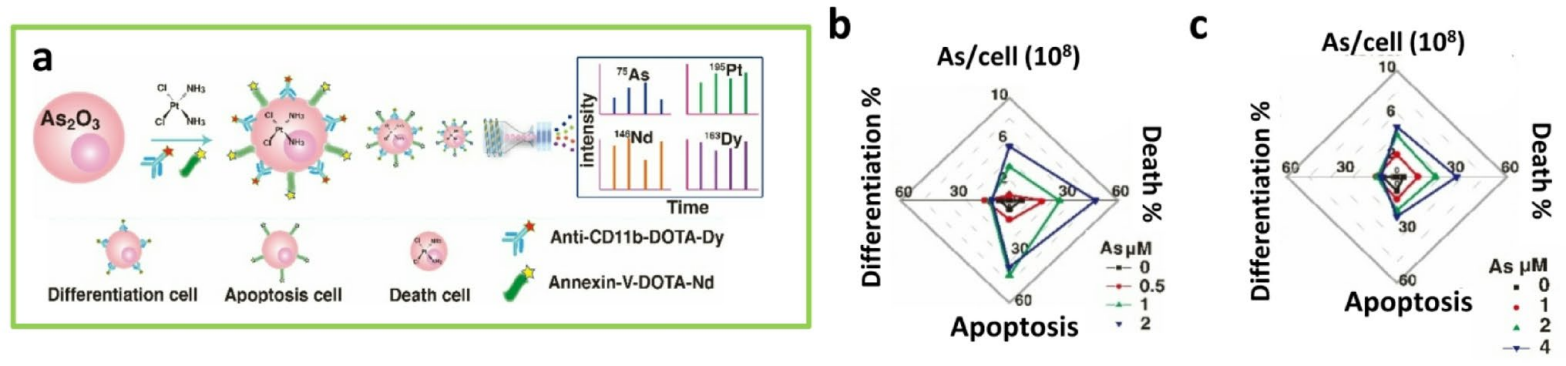
(**a**). Workflow for monitoring arsenic uptake and exploration of As_2_O_3_ therapeutic effects in single leukemia cell by time-resolved ICP-MS. Radar diagrams showing the relationship among intracellular arsenic concentration, cell viability, apoptosis and differentiation ratio of NB4 (**b**) and HL60 (**c**). The figure was redrawn with permission from ref111. Copyright 2017 the Royal Society of Chemistry

Single cell analysis of ATO uptake and cytotoxicity has been achieved using time-resolved ICP-MS with cisplatin as a viability dye and lanthanide-tagged antibodies targeting differentiation (anti-CD11b, conjugated to ^146^Nd) and apoptosis (Annexin-V, conjugated to ^163^Dy) markers (Fig. 9) [114]. By precisely controlling the sampling rate and integration time, arsenic content in individual leukemia cells (NB4 and HL60) was monitored. A radar diagram illustrating the relationship between intracellular arsenic levels and ratios of cell death, apoptosis and differentiation revealed that As_2_O_3_ predominantly induces apoptosis in both NB4 and HL6 with minimal differentiation (Fig. 9), suggesting that apoptosis is primary mechanism of ATO-mediated cell death in leukemia.

Furthermore, the uptake and cytotoxicity of ATO and ZIO-101 across the cell cycle in NB4 and HL60 were evaluated using a double thymidine block combined with time-resolved ICP-MS [115]. Both ATO and ZIO-101 exhibited the highest uptake in the G2/M phase, and lowest in the S phase, with this variation more pronounced for ATO. The expression of key transporters, aquaporin-9 (AQ9) for ATO and xCT for ZIO-101was also cell cycle-dependent [116, 117]. AQ9 expression peaked in the G1 phase, and was lowest in the S phase, while xCT expression was highest in the G2/M phase and lowest in the G1 phase. These transporter expression patterns only partially explained the observed uptake variations. Notably, the cytotoxicity of both ZIO-101 and ATO was cell cycle-dependent with the highest cytotoxicities in the S and G2/M phases and the lowest in the G1 and S phases [115]. These findings underscore the importance of considering cell cycle phases (and probably circadian rhythms) when evaluating the cellular uptake and cytotoxicity of anticancer drugs.

## Conclusion and future perspectives

Proteome-wide identification of arsenic-binding proteins in cancer cells establishes a foundation for elucidating the molecular mechanisms of arsenic-based anticancer drugs from a systems perspective. The thiolphilic nature of arsenic presents challenges in comprehensively tracking the arsenic proteome. Although various metalloproteomic approaches, such as IMAC, GE-ICP-MS, biotinylated arsenical pull-down, and fluorescence-based metalloproteomics, have been employed, each method has limitations. For instance, GE-ICP-MS effectively identifies proteins with strong arsenic-binding affinity but may miss those with low abundance or weak binding. Conversely, fluorescence-based metalloproteomics is limited to detecting proteins with relatively high abundance. Thereby, developing more sensitive fluorescence probes than As-AC by conjugating arsenic-moiety with different fluorophores, may allow identifying more arsenic-binding proteins in cancer cells. Alternatively, methods like thermal proteome profiling (TPP) may be applicable for tracking arsenic-proteome. TPP enables proteome-wide analysis of drug-target interactions, protein-protein interactions (PPI), and post-translational modifications [118] and has successfully identified Bi³⁺-binding proteins in bacterial cells [59]. However, all these techniques possess inherent limitations by the requirement for cell lysis, which can disrupt the native intracellular distribution of arsenic and its interaction with biomolecules. Lysis introduces artifacts, such as redistribution of loosely bound arsenic species, loss of compartmentalization and alterations in protein conformations or redox states that may lead to false-positive or false-negative identification of binding partners.

To overcome these limitations and preserve the spatial context of arsenic distribution, non-destructive imaging techniques like synchrotron-based X-ray fluorescence (XRF) microscopy can be employed [119]. These techniques have been instrumental in advancing the understanding of cellular metabolism of arsenic(As) compounds by enabling high-resolution elemental mapping and in situ speciation analysis in biological samples. In a seminal study on HepG2 human liver cells exposed to arsenite or arsenate, synchrotron microprobe XRF combined with X-ray absorption near-edge spectroscopy (XANES) and extended X-ray absorption fine structure (EXAFS) mapped As accumulation in the euchromatin region of the nucleus predominantly as As-tris-sulfur species, implicating interactions with nuclear proteins (rather than direct DNA binding) as a key mechanism of As-induced toxicity [1]. Similarly, human ovarian IGROV1 cells exposed to inorganic arsenic show consistent As oxidation states across subcellular compartments (nucleus, mitochondria, cytoplasm) with absorption edges indicative of a mixture of species, including potential methylation into organometallic forms that target organelles like mitochondria [120]. In therapeutic contexts, synchrotron radiation X-ray fluorescence (SR-XRF) has supported localization studies of arsenic in leukemia cells treated with optimized As-peptide complexes, aiding the design of tumor-selective delivery systems that exploit cellular uptake pathways to enhance efficacy while minimizing off-target toxicity [9]. Although synchrotron µ-XRF/XAS provides unique, high-resolution spatial and speciation information unmatched by other methods, it has drawbacks including radiation damage, lack of protein-level specificity, low sensitivity for trace interactions, and low throughput, which severely limits its utility as a primary tool for comprehensive As-proteome profiling. It is best used as a targeted, complementary technique after initial discovery of As-proteomes by aforementioned metalloproteomic approaches.

The selective efficacy of arsenic trioxide (ATO) against specific cancer cell lines, such as lymphoma, remains poorly understood. The methodologies developed for ATO can be adapted to investigate the molecular mechanisms of ATO and other arsenic-based drugs across diverse cancer type as well as toxicology of arsenic, facilitating the identification of therapeutic biomarkers. In conclusion, ATO is a promising anticancer agent with multifaceted mechanisms of action. Continued research into its molecular targets at a system level will enable the design of more effective arsenic-based anticancer drugs.

## Data Availability

No datasets were generated or analysed during the current study.
